# Dental Students' Positive and Negative Views on Aging at an Undergraduate Course at the University of British Columbia, Canada

**DOI:** 10.1155/2023/8278510

**Published:** 2023-03-30

**Authors:** Mario Brondani, Adriana B. Siqueira, Renata S. Grazziotin, Diego Ardenghi, Nikolaos Christidis, Antonio B. Siqueira

**Affiliations:** ^1^Faculty of Dentistry, University of British Columbia, 116/2199 Wesbook Mall, Vancouver, BC, Canada V6T 1Z3; ^2^Speech Language Pathologist, Private Practice, 204/2024, Cavalhada Avenue, Porto Alegre 91750-460, Brazil; ^3^Universitetslektor Övertandläkare Ledamot av Karolinska Institutets, Universitetstandvården, Karolinska Institutet, Alfred Nobels Allé 8, Huddinge 14104, Sweden; ^4^Faculty of Chemical Engineering, The Centro Universitário Ritter dos Reis – UniRitter – Campus Zona Sul, Rua Orfanotrófio, 555, Alto - Teresópolis, Porto Alegre, RS 90840-440, Brazil

## Abstract

**Background:**

Different views of aging exist, including ageism as the stereotyped idea about older adults in general. The objective of this study was to perform an initial exploration on how third-year undergraduate dental students at the University of British Columbia (UBC), Vancouver, Canada self-perceived themselves at ages 65, 75, or 85 years old.

**Methods:**

For a period of 10 years, and using a cross-sectional design, third-year undergraduate dental students were asked to envision themselves at 65, 75, or 85 years old in a brief 150-word written essay. We employed a thematic analysis of the textual data via a coding process as suggested. The main themes were identified and displayed in a table with their respective categories of information. NVivo® 12 (QSR International) software was used for data analysis.

**Results:**

Between 2010 and 2020, a total of 519 students enrolled in the UBC undergraduate dental program; 425 essays were collected. Initial coding and thematic analysis led to the identification of four main themes and eight categories; themes included “Ups and Downs,” “Financial Sustainability,” while categories focused on “Trade-offs,” and “Camaraderie,” for example. Different levels of ageism were also apparent when students saw themselves experiencing isolation and forgetfulness regardless of the selected age and the academic year. For other students, they anticipate aging would be healthy, joyful, and productive.

**Conclusion:**

Ageism was present when students imagined themselves at three different older ages, but so were more realistic views of an aging. Further studies are warranted to unravel the impact of geriatric education in tackling stereotypes and ageism.

## 1. Introduction

It has been recognized that the older adult population is quite heterogeneous regarding health and well-being, living arrangements, psychosocial status, and so on [1]. This heterogeneity is even more salient when older adults are divided into three age groups: those between 65 and 74 years of age, those between 75 and 84 years of age, and those older than 85 years of age [2]. The Baby Boomers, referring to those born between 1946 and 1965 and who are now between the ages of 56 and 75, are the fastest growing segment of the population across the globe. And in countries like Canada, they already represent one in every four individuals [3], with those older than 85 years of age growing the most in numbers [4].

Despite aging being predictable and universal physiological phenomena [5], the idea of a permanent youthful-looking body has become the norm fueled by the social and mainstream media, and the cosmetic industry [6] which might foster ageism by implying that aging is something undesirable. Ageism can be defined as “the stereotypes (how we think), prejudice (how we feel) and discrimination (how we act) towards others or oneself based on age” [7], while Butler [8] focused on ageism being against elderly people in particular. In turn, both societal (e.g., structural) and individual level of ageism exist [9], and regardless the age, stereotypes, discrimination, and prejudice are learned and are not innate, and negatively influence the way we feel, perceive, and behave toward others. Among the elderly population in particular, these ageists' attitudes may lead to significantly worse health psychological outcomes, poor social connections, lack of working opportunities [10], and ultimately impacting quality of life and well-being negatively [11].

In order to reduce or eliminate ageism altogether, efforts have been made to support educational activities aimed at reducing prejudice and dispelling misconceptions about older adults, and demystifying stereotypical views of aging [12]. Some of these activities include promoting meaningful interactions with older adults, critically reflecting about such interactions [13–15], and normalizing aging as part of being human to foster critical thinking and empathy toward older adults [16]; these activities may take place in earlier education and/or in postsecondary training such as within a dental geriatric course involving university undergraduate students. This study explored the extent to which undergraduate dental students at the University of British Columbia (UBC) in Vancouver, Canada, hold ageist views about aging when asked to envision themselves at a given old age. Ethics was received from the UBC Behavioural Research Ethics Board (# H19-01005).

## 2. Methods

A generic descriptive interpretative qualitative inquiry [17] was employed to explore thematically written short essays submitted between 2010 and 2020 by undergraduate third-year dental students at UBC's Faculty of Dentistry.

For over a decade, UBC's Faculty of Dentistry has offered a 2-year undergraduate course for third- and fourth-year students in dental geriatrics with didactic and experiential activities [18–20], including interactions with older adults in a clinical context and in nursing homes [13]. This 2-year dental geriatric course runs for the entire academic year, from September to May, during the last 2 years of the 4-year dentistry program. The course covers a number of topics and uses a variety of teaching methodologies [13, 21, 22]. For the first 5 min of the first session of the first year of this course, third-year dental students are asked to write about “how do I see myself at the age of 65… (or) …75 (or)… 85” as they reflect freely on any topic or issue about getting older and aging, and before they receive any instruction or information about dental geriatrics or caring for older adults in this course.

Students are asked to individually write a short essay on the specific age assigned by the course instructor who randomly distributes a blank card with one of the three ages printed on the card. Students are informed that they should not use more than 150 words on the essay while envisioning themselves at that specific age. As students do not receive any information about format or content of this activity, they may complete the essay by referring to their observations and interactions (or lack thereof) with older adults and/or elderly family members, and/or from what they read and observe on the media, including TV shows, newspapers, advertisements, news outlets, websites, podcasts, social media platforms, and so on. Students are free to write about any positive or negative view they might hold in terms of oral and general health status, body functions, psychosocial conditions, mental status, familial arrangements, housing situations, personal and professional relationships, and so on. As a free-format activity, there is no need to cite references to support or refute their views.

This written short essay has been part of the dental geriatric course since 2009, was never assessed or analyzed for content, it is not mandatory to complete it, and it is not graded; on this manuscript, we performed an initial analysis on the essays submitted from 2010 to 2020 only, not including the pandemic years. The age range for third-year dental students at UBC varies between 23 and 31 years old, from all genders. Students completed the essay in class using pen and paper. Gender, age, or any other identifiers were not available.

### 2.1. Data Analysis

**Figure 1:**
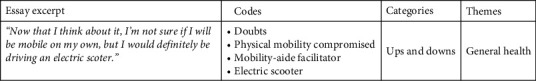
Coding scheme using an excerpt from one of the essays.

NVivo12® software from QSR International was used for sorting, organizing, and analyzing the essays textual information. We utilized an inductive coding process to develop categories and themes from the essays as suggested by Braun and Clarke [23]. More specifically, as we have previously described [13, 24, 14, 25], we started by reading all the essays to get familiar with their overall content. We then proceed by systematically coding all the essays and generating initial themes from these codes. The coding process helps to identify ideas in the form of a word or short phrase in the write-ups. These words or short phrases—the codes—were later grouped into categories expressing similar thoughts; we proceeded to find the relationships between these categories, which together composed a main theme [13, 15, 25] (Figure 1). We selected four main themes as presented in this manuscript. This process allowed us to reach saturation, when no new information was emerging from the various essays [26]. Two authors (M.B. and A.S.) interactively analyzed all the essays together and developed an initial coding scheme. As the two authors continued to build the coding scheme, they met via conference calls twice a week, between January 1 and March 10, 2020, discussing disagreements until consensus.

## 3. Results

The UBC's Faculty of Dentistry class size varies every year and between 2010 and 2020, we were able to gather 425 essays (82%) out of all 519 students enrolled during those 10 years; from the 94 remaining students, 66 did not participate (likely given that the essay was voluntary and not graded) and 28 were not present on that first day of classes. The inductive coding and thematic analysis of the 425 short essays led to 372 codes (data are not shown), eight categories, and four main themes (Table 1). The main themes likely overlap in meaning and significance, but they are shown separately for better visualization and comprehension. We also used selected excerpts from the essays representing the three age groups to exemplify categories and themes. These excerpts are neither exhaustive nor representative of all 425 essays. We have not provided the frequencies that a given code or category showed up because such frequencies might imply importance or those less frequently identified.

## 4. Discussion

In this study, we explored the extent to which undergraduate dental students at UBC hold ageist views about aging when asked to envision themselves at age 65, 75, or 85. Structural- and individual-level ageism were present mostly when students wrote about stereotypical views on getting older aging (“annoying my family with repetitive questions …”; “have some senior moments, and perhaps forget things and repeat myself a lot”) and even prejudice (“…job at my age – who would hire me?”; “no family, isolated, likely in a nursing home”) (Table 1). Discrimination per se did not surface directly as it usually refers to how one acts—behaves—toward somebody else and students might not have written about that.

Considering the three ages (65, 75, and 85) and the entire sample (from 2010 to 2020), a number of students expressed negative views about aging filled with physical dependency on others, marital disarray, edentulism, and mental deficits (Table 1).

But for many other students, aging or being an older adult was understood within the expectations of feasible retirement, companionship, a functional dentition, and active and connected lifestyles across the three assigned ages (Table 1). For the vast majority of the essays (*n* = 398, 94%), students wrote both positive and negative ideas about their assigned ages in relation to a particular area (e.g., living alone and taking multiple medications, but receiving a high pension and having all natural teeth). As a result, tabulation of percentages or the frequency at which a given view was mentioned does not lead to meaningful information when comparing cohorts. In fact, over the 10-year period that the short essays were collected, we did not observe any variation in terms of the various self-perceived ideas about the three assigned age groups.

It was clear from the essays that although not formally receiving any didactic material about aging, ageism, and age-related changes before this activity, ageist ideas were shared to the same extent as more positive views of aging. For example, numerous students referred to potential memory loss, cognitive decline, and forgetfulness, as examples of individual-level ageism. It is known that as memory declines even in normal aging [27], it is crucial to not label every concern related to forgetfulness in older adults as necessarily negative [28]. At the same time, many students mentioned themselves still riding scooters, solving puzzles, and reading, which are activities suggested to be performed given the positive influence on cognition and brain health [15].

Some students imagined themselves alone and unemployable, which might be a reality for some but not others [12]. When social connectedness is seen at an individual-level ageism, older adults tend to experience less social support and engagement, and feel isolated [25]. In terms of the workforce engaging older adults as hires, the lack of work opportunities, as an example of structural-level ageism, may lead to depression [12].

No student wrote about being denied access to health care, which would represent a clear form of structural ageism [10]. However, health care system in countries like Canada [29] and the USA [30] continues to be unprepared for the complexities of caring for some older adults [31–33]. Although these studies do not mention denial of care, longer waiting times for an appointment may be the norm in a health care crisis, which further contributes to worsening general health [34]. On the contrary, many students saw themselves still active and functionally fit as they age, which corroborates the call for maintaining physical activity in order to promote successful aging among older adults [35].

Individual-level ageism surfaced when many students imagined themselves dead at any of the three ages. Although death is part of being alive, and life expectancy does decrease as we age, older adults from developed countries at ages 65, 75, and 85 are expected to live for at least 22, 14, and 8 more years, respectively, despite some older adults finding themselves living with disability and illness—the ups and downs in life [36].

Within some negative and perhaps stereotyped views about aging, many students did emphasize normal expectations that come with growing older, including retirement, changes in family relationships, and setbacks in general health. As many governments have questioned the idea of retirement at 65 [37]—the age used as a benchmark for most pension plan calculations—some students did envision themselves still working if not forced to retire due to working-related repetitive motion injuries, or health concerns [38].

Students expressed a variety of family relationships at their assigned ages: some would be living alone or with pets, others would be living with their spouses and family members, and others would be living in isolation. It is known that keeping family relations in old age correlates with happiness, a sense of coherence, and good health [39]. On the contrary, isolation and loneliness may lead to negative quality of life and even premature death if no intervention is taken [40].

As the self-perceived general health at any of the three ages fluctuated throughout the essays, so did the self-perceived oral health as some students would expect to be edentulous, while others would have a functional dentition. Although some students thought about their grandparents' oral health to reflect about their own dentition, loosing teeth as we age is mostly due to dental caries and not aging per se [41].

Students' self-perceptions of themselves at three different ages were both positive and negative; while some expressed misconceptions, others might have been ageist against their own selves. In turn, it remains important to educate future health care providers about normal and pathological aging, give them opportunities for intergenerational activities, and normalize aging. Dental geriatrics courses like the one within UBC's Faculty of Dentistry may help to reduce prejudice and stereotypes, and demystify misconceptions when they openly discuss normal and pathological aging, expose students to various living arrangements of older adults, bring the elderly to the classroom, and foster the idea of successful aging [42].

Although informative, this study has limitations. There was no validation of the question posed to guide the brief essays [43]. The information presented herein came from specific cohorts of undergraduate students who live in a specific country so their views on aging and older adults cannot be generalized because the quality of life, especially for the elderly, is different around the world. The cross-sectional nature of the study does not allow for comparison before and after the geriatric course, for example. The thematic analysis is limited and not contextualized due to the lack of information on students' age, gender, cultural backgrounds, and family living arrangements. Response bias might have happened as students might have written what they thought the course instructor would like to read rather them their own reasoning and ideas. As an initial thematic exploration, the themes and categories that emerged are not exhaustive of all 425 essays, do not represent all viewpoints about aging, and might overlap in meaning and relevance; however, saturation was reached given the themes and categories presented. A more in-deep inductive and thematic analysis of the existing essays and the ones submitted during the COVID-19 pandemic years is suggested. Further studies are warranted to unravel the impact of geriatric education in tackling stereotypes and ageism.

## 5. Conclusion

The initial coding and thematic analysis of 425 essays revealed the presence of structural- and individual-level ageism views when undergraduate dental students were asked to think about themselves at one of the three different older ages, 65, 75, or 85 years old. However, more realistic and less pessimistic views of aging were also considered. It is unknown if ageist ideas would decrease and less pessimistic outlook about aging would increase after students experience the 2 years of the geriatric course. More in-deep studies of the essays are needed and further research is warranted to unravel the impact of geriatric education in tackling stereotypes and ageism.

## Figures and Tables

**Table 1:** Themes, categories, and excerpts from 521 student write-ups about aging expectations at 65, 75, and 85 years old.

Themes	Category	Excerpt
Ups and downs	Trade-off general health	“I see myself completing puzzles and crosswords, but forgetting about what I had for dinner the night before” (Age 85, 2019). “trying to face-time with my friends but not knowing how to operate my pad” (Age 75, 2015). “talking with my grandchild and asking myself – who is this lovely boy?” (Age 75, 2010). “annoying my family with repetitive questions while being the favourite grandpa” (Age 85, 2017). “having problems with arthritis, but being able to still keep reading my favourite novels” (Age 75, 2018). “I would be doing aquafit so with my frail knees get a rest” (Age 75, 2013). “not sure if I will be mobile on my own, but I would definitely be driving an electric scoter” (Age 85, 2012).

Financial sustainability	(Un) winding down	“working and still enjoying it.” (Age 65, 2014). “as a dentist, would be retired due to my back – it already hurts!” (Age 75, 2019). “retired? Only with enough income so I do retire and have not need to find a job at my age – who would hire me?” (Age 85, 2017). “hope to be retiring soon, or be already retired, with financial stability” (Age 65, 2020). “like my daddy's dad, I would be still working at 75, with a sharp mind and good bank account” (Age 75, 2014). “how much do I need to retire? Will an annual income of 20 k be enough? I hope so as I also hope to have no debt” (Age 75, 2010).

Global health	Mind, body, and soul A mouthful The World's End	“will I remember I did this activity in class back in 2012? I doubt it” (Age 65, 2012). “looking at my grandma, I think I will be as sharp as ever” (Age 75, 2015). “I get a bit frustrated in talking with my grandpa as he forgets a lot of things and I have to repeat myself – I hope my grandkids do not feel the same way” (Age 85, 2019). “feeling happy and smiling at simple things in life” (Age 65, 2011). “recollecting my adventures and world explorations and wanting more” (Age 85, 2015). “as they say, I might have some ‘senior moments' and perhaps forget thinks, repeat myself a lot, annoying others” (Age 75, 2020). “brushing regularly, not really flossing – only when food get stuck in between my teeth” (Age 85, 2011). “perhaps no teeth, when I look at my grandpa, and not sure about wearing dentures either” (Age 85, 2014). “no reason to have lost any of my teeth - I do not have any cavities now” (Age 75, 2015). “some teeth might have been broken off, but will likely have a good dentition” (Age 65, 2019). “mild gingivitis, no pain, some dental work to be done but not an emergency” (Age 65, 2012). “not here anymore” (Age 85, 2020). “given my genetics, will still be alive and kicking” (Age 75, 2018). “finally getting flowers from my children … on my tombstone” (Age 75, 2019). “long dead” (Age 85, 2012). “isn't 75 the new 50's? If so, will be enjoying my retirement” (Age 75, 2017).

Me, myself, and I	Familial relationships camaraderie	“living on my own, independent, and working around the garden” (Age 85, 2011). “having my house shared with my family and their families” (Age 75, 2012). “I'm a cat person. I hope to have two cats snuggling with me” (Age 65, 2019). “enjoying a retirement community, with nothing to worry about” (Age 75, 2016). “no family, isolated, likely in a nursing home” (Age 85, 2018). “living with my life partner, being loved, feeling loved, and loving … but no kids” (Age 75, 2017). “still connected with the community, and volunteering” (Age 65, 2015). “placed in a nursing home and my wife in another, separated” (Age 65, 2015). “getting some help from neighbours and friends” (Age 75, 2017). “volunteering at my church, having communal activities, feeling connected” (Age 85, 2012). “happily living on my own, no kids, nobody to bother me” (Age 85, 2013). “married for the second (or third time)… what can I say, I'm a good catch and a friendly company” (Age 65, 2014). “not sure, but I would expect visiting my closest friends who are still alive and not talking about death!” (Age 75, 2019). “being married twice, both times for love” (Age 65, 2019). “living with a lover at a gated retirement facility” (Age 75, 2010).

## Data Availability

Due to the sensitivity of some of the information shared via the assignments, only deidentified data can be made available upon request to the first author.
